# Intraventricular cystic papillary meningioma: A case report and literature review

**DOI:** 10.1097/MD.0000000000021514

**Published:** 2020-07-31

**Authors:** Zhe Cheng, Qing Chao, Hui Zhang, Da-Wei Wang, Han-Sheng Shu

**Affiliations:** Department of Neurosurgery, The Second Affiliated Hospital of Bengbu Medical College, China.

**Keywords:** cystic, intraventricular, papillary meningioma, prognosis, treatment

## Abstract

**Introduction:**

Papillary meningioma is an extremely rare malignant lesion with high degree of invasiveness, high recurrence rate, and perivascular pseudopapillary structure. The incidence of cystic degeneration in papillary meningiomas is relatively low, and cystic papillary meningiomas growing in the ventricle are even rarer. Here, we present a case of cystic meningioma and review the literature to propose the diagnosis, treatment, immunohistochemical features, and prognosis of the same.

**Patient concerns:**

In July 2013, a 35-year-old male Chinese patient presented with dizziness that lasted for a week, without relief. Magnetic resonance imaging (MRI) revealed a 2.0 cm × 1.5 cm × 3.0 cm-sized mass located in the left lateral ventricle trigone. The tumor was small and likely non-malignant. Therefore, the patient received conservative treatment and regular follow-ups. In June 2017, the patient experienced sudden severe headache, dizziness, and vomiting.

**Diagnosis and intervention:**

MRI revealed that the mass in the left lateral ventricle trigone had increased to 5.0 cm × 7.0 cm × 8.0 cm over 4 years. The patient underwent surgical resection via the left parietal–occipital approach. Two months postoperatively, the patient received 60 Gy local radiotherapy. The postoperative histopathology suggested that the mass was a cystic papillary meningioma.

**Outcomes:**

Two years after the operation, the patient was asymptomatic, and no recurrence of the lesion was noted on MRI.

**Conclusion:**

The diagnosis of intraventricular cystic papillary meningioma depends mainly on its histology and imaging features. Total resection and adjuvant radiotherapy can result in a relatively good prognosis of patients with intraventricular cystic papillary meningiomas.

## 1 Introduction

Meningiomas are the most common intracranial tumors in adults, which originate from arachnoid cap cells, and account for approximately 20% to 36% of all primary intracranial tumors.^[1–4]^ Papillary meningiomas are a special type of meningiomas, which are considered grade III malignant tumors with a very low overall incidence, according to the World Health Organization (WHO) classification.^[3,5]^ Papillary meningiomas exhibit characteristic features, such as high degree of invasiveness, high risk of postoperative recurrence, and poor prognosis.^[2,6]^ They are histologically characterized by the growth of perivascular pseudopapillary tumor cells.^[7,8]^

Most papillary meningiomas grow in the supratentorial, foramen magnum, vertebral canal, and petrous regions,^[9,10]^ but rarely in the ventricle. Zhi et al^[11]^ reported the first case of a cystic papillary meningioma in the lateral ventricle that invaded the brain parenchyma, in which resection and local radiotherapy achieved good results. There is limited knowledge about the mechanism of cystic formation in papillary meningiomas, and few therapeutic experiences are available for reference. Therefore, we reviewed the literature and summarized several successful cases.

In this study, we report a rare case of cystic papillary meningioma growing in the left lateral ventricle trigone and perform a literature review. The epidemiological characteristics, diagnosis, treatment, and prognosis of papillary meningiomas are systematically summarized in this paper.

## 2 Case presentation

**Figure 1:**
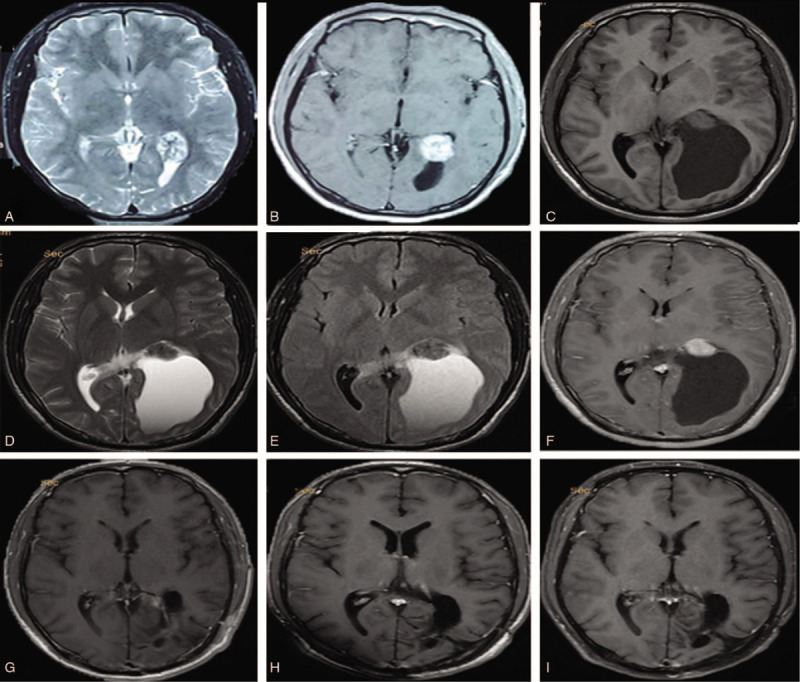
Magnetic resonance imaging revealing a cystic-solid mass in the left lateral ventricle trigone. (A) Magnetic resonance imaging (MRI) T2 sequence was performed on July 10, 2013. (B) Enhanced MRI was performed on August 12, 2014, which revealed that the tumor parenchyma was homogeneously enhanced. (C–F) MRI was performed 1 wk before operation on July 29, 2017, which suggested cystic changes in the tumor (C: T1; D: T2; E: T2-FLAIR; F: enhanced). (G–I) Postoperative enhanced MRI showed no tumor growth (G: 6 mo after surgery; H: 1 yr after surgery; I: 2 yr after surgery).

In July 2013, a 35-year-old Chinese male presented with dizziness that had lasted for a week without relief. At that time, magnetic resonance imaging (MRI) revealed a mass, sized 2.0 cm × 1.5 cm × 3.0 cm, located in the left lateral ventricle trigone (Fig. 1A). The clinical symptoms were atypical and relieved, and the tumor was small and likely non-malignant. Therefore, the patient received conservative treatment and regular follow-ups. He irregularly underwent routine cranial MRI examinations to dynamically observe tumor growth; however, no obvious growth of the tumor was found except for a slight increase in the size of the cyst (Fig. 1B).

In June 2017, the patient suddenly suffered from severe headache, dizziness, and vomiting. Cranial MRI showed that the tumor in the left ventricle trigone developed into a cystic-solid mass, which increased to 5.0 cm × 7.0 cm × 8.0 cm, mainly due to an increase of cystic fluid (Fig. 1C–E). Enhanced MRI revealed a homogeneous enhancement of the tumor parenchyma; however, no enhancement of the cystic fluid or tumor wall was noted (Fig. 1F). The tumor cystic fluid showed hyperintense signals on T2-weighted-fluid-attenuated inversion recovery (T2-FLAIR) sequences, suggesting that the cyst did not communicate with the ventricle (Fig. 1D and E). The ventricle was small, and there was no sign of hydrocephalus. We were inclined to diagnose a choroid plexus tumor before operation. In order to improve the symptoms and confirm the pathology, the patient underwent surgical resection via the left parietal–occipital approach. During surgery, we found that the boundary of the tumor was unclear, invaded the surrounding brain tissue, and firmly adhered to the ventricular wall but was separated from the ventricular system by a layer of membrane. The first step was to release the cystic fluid, which was followed by separation of the cystic wall as far as possible along these boundaries, and finally total resection of the solid part of the tumor. The cystic fluid was serous and xanthochromic and was considered to be secreted by tumor cells or liquefied after intratumoral hemorrhage. Postoperative MRI scans confirmed that the tumor had been completely removed (Fig. 1G).

**Figure 2:**
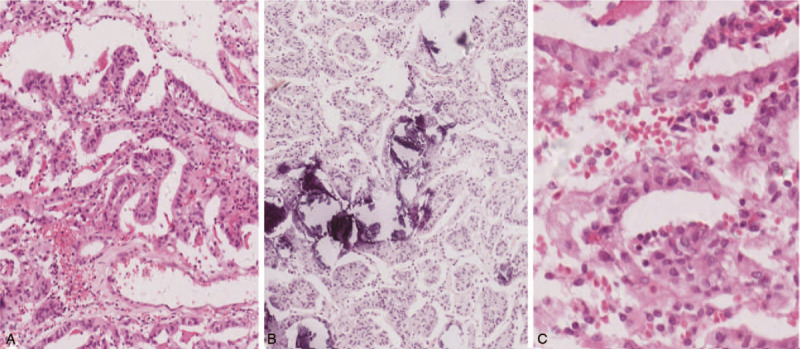
Hematoxylin and eosin staining. (A and C) The tumor consisted of a large number of monolayer columnar cells, arranged in a predominantly papillary pattern with perivascular pseudorosettes. (A) Original magnification 100×, (C) original magnification 400×. (B) Psammoma body structure in tumor cells. Original magnification 100×.

**Figure 3:**
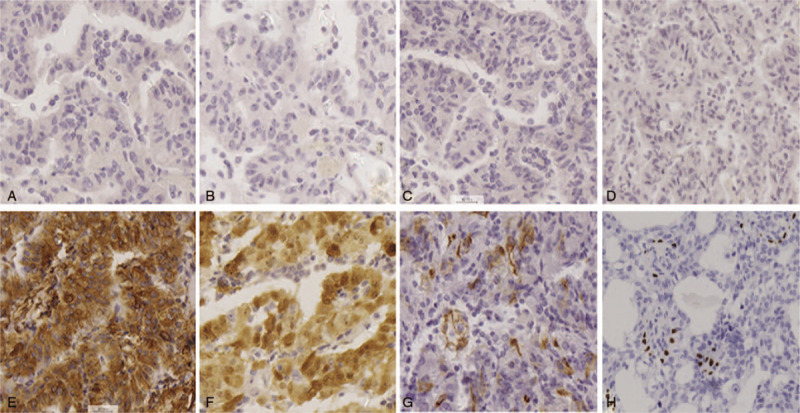
Immunohistochemical staining. Immunohistochemistry demonstrated negativity for EMA (A), GFAP (B), CK-7 (C), and TTF-1(D) but positivity for vimentin (E), S-100(F), CK (G), and ki-67(H). Original magnification 400×.

Histological examination showed that the tumor tissue was composed of multilayer cells arranged in a predominantly papillary pattern with perivascular pseudorosettes and tumor cells with psammoma bodies (Fig. 2). Immunohistochemical staining revealed that both vimentin and S-100 protein were strongly overexpressed, and that cytokeratin was diffusely positive, while glial fibrillary acidic protein (GFAP), cytokeratin-7 (CK-7), thyroid transcription factor-1 (TTF-1), and epithelial membrane antigen (EMA) were negative. The expression level of the Ki-67 antigen (MIB-1) was as high as 5% (Fig. 3). Therefore, the patient was diagnosed with cystic papillary meningioma (WHO III). Histopathological specimens were reviewed by at least 3 experienced pathologists. Two months postoperatively, the patient received 60 Gy local radiotherapy. One year after radiotherapy, the patient was asymptomatic, and no recurrence was noted on MRI (Fig. 1H). Two years postoperatively, the enhanced MRI showed no tumor growth (Fig. 1I). The patient is currently undergoing routine follow-ups.

## 3 Discussion

Papillary meningiomas often exhibit malignant features associated with a poor prognosis, including high likelihood of aggressive behavior and frequent recurrence.^[12]^ Cystic meningiomas are rare variants of meningiomas, and cystic papillary meningiomas that are located in the ventricle are extremely rare. To the best of our knowledge, only 1 case of intraventricular cystic papillary meningioma has been reported in the literature; hence, there is a lack of clinical treatment experience available for reference.

In order to summarize the characteristics of cystic papillary meningiomas, we performed a literature review of studies published in recent decades by searching PubMed, MEDLINE, and EMBASE, as well as published conference proceedings. We used the following key words for our search strategy: “meningioma,” “papillary,” “papillary meningioma,” “cystic papillary meningioma,” “intraventricular,” “treatment,” and “prognosis.” Seven cases, including the present one, were of cystic papillary meningiomas.

Several characteristics of cystic papillary meningiomas have been summarized from the literature (Table 1). Cystic degeneration of papillary meningiomasis extremely rare, and the specific mechanisms of cystic formation are still unclear.^[13,14]^ Wakabayashi et al concluded that the formation of a cyst may be one of the characteristics of papillary meningiomas.^[15]^ According to the color of the cystic fluid, our view is that the process of cystic degeneration is related to the secretion of tumor cells or microbleeding in the tumor. Cystic papillary meningiomas are more common in women^[16]^ and young patients; they have also been reported in children.^[17,18]^ Papillary meningiomas have exceedingly abundant blood supplies and aggressively invade into the adjacent brain. Papillary meningiomas are histopathologically characterized by the growth of perivascular pseudopapillary tumor cells, necrotic areas, and increased mitotic activity of tumor cells. Total resection with or without radiotherapy is highly recommended as the best treatment along with timely follow-ups.^[11,19]^

**Table 1:**
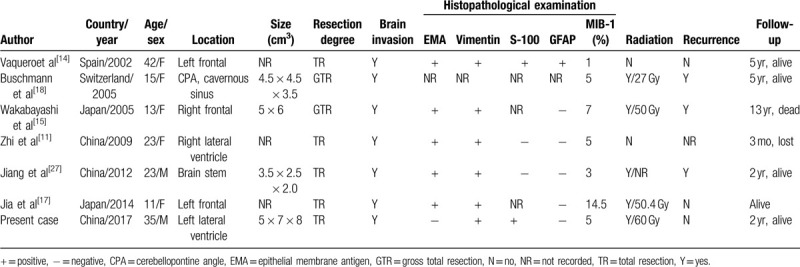
Characteristics of cystic papillary menigiomas reported in literature and present.

Papillary meningiomas are mostly found in the supratentorial compartment,^[9]^ and rarely in the posterior fossa, spinal canal, ventricle, or jugular foramen region.^[20,21]^ However, the location of cystic papillary meningioma growth is not very regular. A rare case of intraventricular cystic papillary meningioma was reported, which did not, however, serve as a reference for diagnosis and treatment, as the patient passed away 3 months after surgery.^[11]^ In our present case, the tumor was located in the left lateral ventricle and was initially diagnosed as a choroid plexus tumor. Intracranial papillary meningiomas are often misdiagnosed as choroid plexus papillomas and their differentiation can be very difficult. Choroid plexus tumors, including choroid plexus papillomas and choroid plexus papillary carcinomas, originate from choroid plexus epithelium and usually lead to communicating hydrocephalus due to abnormal secretion and absorption of cerebrospinal fluid.^[22]^ In our patient, there was no enlargement of the ventricular system and no occurrence of hydrocephalus. This is an indirect indication for excluding a choroid plexus tumor. Pathological manifestations play a decisive role in differential diagnosis. Immunohistochemical characteristics of choroid plexus papillomas are positive expressions of both cytokeratin and vimentin, while transthyretin and S-100 protein are expressed in 80% to 90% of choroid plexus carcinomas. A focal distribution of GFAP is observed in 25% to 55% of choroid plexus papillomas and 20% of choroid plexus carcinomas.^[23]^

The immunohistochemical results of our patient showed that vimentin and S-100 protein were both strongly overexpressed, cytokeratin was diffusely positive, and ki-67 expression was up to 5%, while EMA, GFAP, CK-7, cytokeratin-20 (CK-20), and TTF-1 were negative. Generally, the expression of EMA is positive in >90% of benign meningiomas and <20% of malignant meningiomas. Thus, the negative expression of EMA in our patient indicates a malignant meningioma.^[24,25]^

MRI T2-FLAIR sequences have revealed that the signal of tumor cystic fluid is slightly higher than that of cerebrospinal fluid and does not participate in cerebrospinal fluid circulation. Our patient exhibited a type 2 cyst according to the Nauta classification system of intracranial cystic meningiomas.^[26]^ The cyst was actually located inside the tumor and was not connected to the ventricle, but its margin was formed by a thin layer of tumor cells. We infer that the rapid growth of the tumor and the abundant blood supply of the meningioma itself may lead to intratumoral hemorrhage, which leads to the cystic degeneration of papillary meningiomas.

The diagnosis of intraventricular cystic papillary meningioma mainly depends on imaging and histopathology. The most typical computed tomography (CT) scan of intraventricular papillary meningiomas usually exhibits high density in the parenchymal and low density in the cystic part. Enhanced imaging usually reveals no enhancement of cystic fluid but an occasional enhancement of the cyst wall of the tumor. Surgical resection is the key treatment for intraventricular cystic papillary meningioma, and total resection with or without radiotherapy is recommended as the standard treatment.^[27]^ Surgical resection of the tumor can not only relieve clinical symptoms but also obtain specimen tissues required for pathological diagnosis. Fong et al^[28]^ provided valuable information for the best therapeutic schedule for patients diagnosed with papillary meningioma, which consists of complete excision together with adjuvant radiotherapy.

In conclusion, we report an unusual cystic papillary meningioma growing in the lateral ventricle. The tumor was completely removed, and the patient subsequently received 60 Gy local adjuvant radiotherapy. The patient recovered well, was uneventful, exhibited no recurrence of the tumor on reexamination, and will continue to undergo follow-up.

## 4 Conclusion

The diagnosis and differential diagnosis of intraventricular cystic papillary meningioma mainly depend on its histology and imaging. The key to the operation is slow release of the cystic fluid to achieve decompression, followed with careful separation of the cyst wall along its boundary and, finally, complete removal of the tumor. Total resection and adjuvant radiotherapy can result in a relatively good prognosis of patients with intraventricular cystic papillary meningiomas. Limited by insufficient basic research and few clinical cases, the mechanism of cystic degeneration of papillary meningiomas is still unclear.

## Author contributions

**Date curation:** Zhe Cheng, Qing Chao, Hui Zhang.

**Funding acquisition:** Zhe Cheng, Han-Sheng Shu.

**Pathological report examination:** Da-Wei Wang, Han-Sheng Shu.

**Surgery performed by:** Zhe Cheng, Da-Wei Wang, Han-Sheng Shu.

**Writing – original draft:** Zhe Cheng, Qing Chao.

**Writing – review & editing:** Da-Wei Wang, Han-Sheng Shu.
